# Raspberry-Like Bismuth Oxychloride on Mesoporous Siliceous Support for Sensitive Electrochemical Stripping Analysis of Cadmium

**DOI:** 10.3390/molecules22050797

**Published:** 2017-05-13

**Authors:** Yiyan Song, Zhihui Xu, Xinyu Yu, Xueyan Shi, Huijun Jiang, Xiaoming Li, Yan Kong, Qin Xu, Jin Chen

**Affiliations:** 1 School of Public Health, Nanjing Medical University, Nanjing 211166, China; yiyansong@njmu.edu.cn (Y.S.); xuzhihui@njmu.edu.cn (Z.X.); kimiyxy@njmu.edu.cn (X.Y.); 2 School of Pharmacy, Nanjing Medical University, Nanjing 211166, China; xueyan_shi0805@163.com (X.S.); huijun_jiang@njmu.edu.cn (H.J.); 3 State Key Laboratory of Materials-Oriented Chemical Engineering, College of Chemistry and Chemical Engineering, Nanjing University of Technology, Nanjing 211166, China; ntulxm@163.com; (X.L.); kongy36@njtech.edu.cn (Y.K.); 4 College of Chemistry and Chemical Engineering, Yangzhou University, Yangzhou 225002, China; xuqin@yzu.edu.cn; 5 The Key Laboratory of Modern Toxicology, Ministry of Education, School of Public Health, Nanjing Medical University, Nanjing 210093, China

**Keywords:** blood cadmium, mesoporous materials, bismuth, anodic stripping voltammetry, ICP-MS

## Abstract

BiOCl-SiO_2_ KIT-6 composite materials with raspberry-like structures are facilely prepared under hydrothermal conditions. The mesoporous siliceous support of SiO_2_ KIT-6-incorporated BiOCl with enlarged yet refined surface morphology characterized by physiochemical methods exhibits an improved electrochemical performance. A sensitive electrochemical detection method of cadmium concentration using square wave anodic stripping voltammetry was developed based on BiOCl-SiO_2_ KIT-6 composite-modified glassy carbon electrodes, which displayed wide linear ranges of 0.5 to 10 μg/L and 10 to 300 μg/L and a detection limit of 65 ng/L. The sensitive, versatile and eco-friendly sensor was successfully applied for the determination of cadmium-spiked human blood samples.

## 1. Introduction

As one of the most distributed heavy metals, cadmium contamination induced by the discharge from automobiles, mining, and agriculture [1] remains an increasingly challenging threat to public health due to its relatively long half-life degradation in the environment and human body. Exposure to cadmium causes adverse effects on kidney function and bone density of humans, resulting in an increased risk of tumorigenesis, such as lung cancer [2,3]. Thus, it is important to search for a rapid, sensitive, and ideally portable analytical tool for the purpose of monitoring cadmium levels on a large scale.

The available standard methods for heavy metal determination include atomic absorption spectrometry [4,5], atomic fluorescence spectrometry [6] and inductively coupled plasma mass spectrometry (ICP-Mass) [7,8,9]. However, these methods heavily rely on professional instrumentations that may not be suitable for the field and on-spot applications. Electrochemical methods that possess qualities of portability, ultra-sensitivity, readiness and low cost [10,11] are useful and efficient methods for the trace determination of heavy metals [12]. In particular, anodic stripping voltammetry (ASV), which involves an effective preconcentration step in addition to sensitive electroanalysis generating a high signal-to-noise ratio, has been employed for such a purpose during the last decade [13,14,15,16,17,18,19,20,21]. Due to the deposition of heavy metals on the sensor surface during the measurement, the chemical components of the modified electrode are closely related to its analytical performance towards target analytes.

Owing to its high photocorrosion stability and good biocompatibility [22,23], bismuth oxychloride (BiOCl), one of the most important bismuth oxyhalides, has wide applications in fields such as cosmetics and photo-catalytic/-electrochemical materials. Meanwhile, bismuth-based modified electrodes such as Bi(NO_3_)_3_ [24,25,26,27,28] and Bi_2_O_3_ [29,30,31] have been used for the electrochemical stripping analysis of heavy metals as a green method in an effort to replace toxic mercury-based sensors. Compared with other bismuth-based materials, the layered structure of BiOCl favors the transfer of photogenerated electrons and holes, while it prohibits the recombination of electron-hole pairs, thus leading to good photocatalytic performance [32,33]. However, the tendency of BiOCl to form a layered structure makes it rather difficult to produce materials with defined morphologies on a large batch basis.

Ordered mesoporous silica materials such as MCM-41, SBA-15 and KIT-6 are attracting more attention due to their promising potential in catalysis, biosensing and drug-delivery [34,35,36,37]. Particularly, SiO_2_ KIT-6 exhibits a three-dimensional (3D, large-pore gyroid cubic *Ia3d*) pore structure with an interpenetrating bicontinuous framework of channels, which is desirable for the structural modifications and incorporation of heteroatoms [38]. Therefore, we set out to synthesize BiOCl-SiO_2_ KIT-6 composite materials aimed at producing BiOCl with a defined morphology using mesoporous KIT-6 supports.

In the present study, we have successfully used the mesoporous siliceous materials of SiO_2_ KIT-6 as structural supports to produce a novel BiOCl-SiO_2_ KIT-6 composite material for the stripping analysis of cadmium. The composite BiOCl-SiO_2_ KIT-6 materials were facilely synthesized in situ, resulting in the defined surface morphology of BiOCl. The produced composite materials were characterized by a combination of physicochemical as well as electrochemical methods. BiOCl-SiO_2_ KIT-6 composite-modified glassy carbon electrodes (BiOCl-SiO_2_ KIT-6/GCEs) were constructed for the sensitive detection of cadmium in solutions and in human blood samples. The decorated sensor using BiOCl-SiO_2_ KIT-6 composites showed a wide calibration concentration range of cadmium from 0.5 to 300 μg/L and a wide working potential window, which may permit the discrimination of multiple heavy metals.

## 2. Results

### 2.1. Morphological Characterization

**Figure 1:**
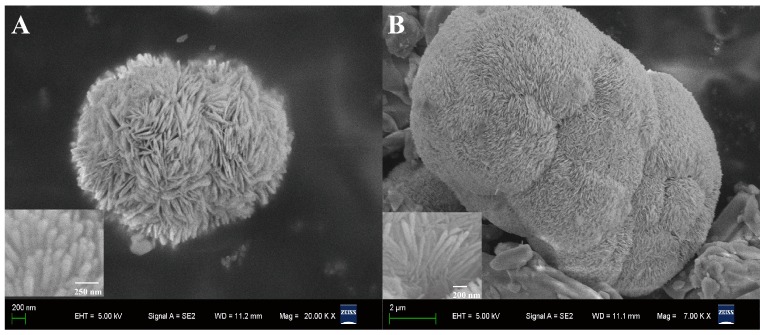
SEM images of (**A**) BiOCl and (**B**) BiOCl-SiO_2_ KIT-6 composite, with insets showing magnified views of the surfaces.

**Figure 2:**
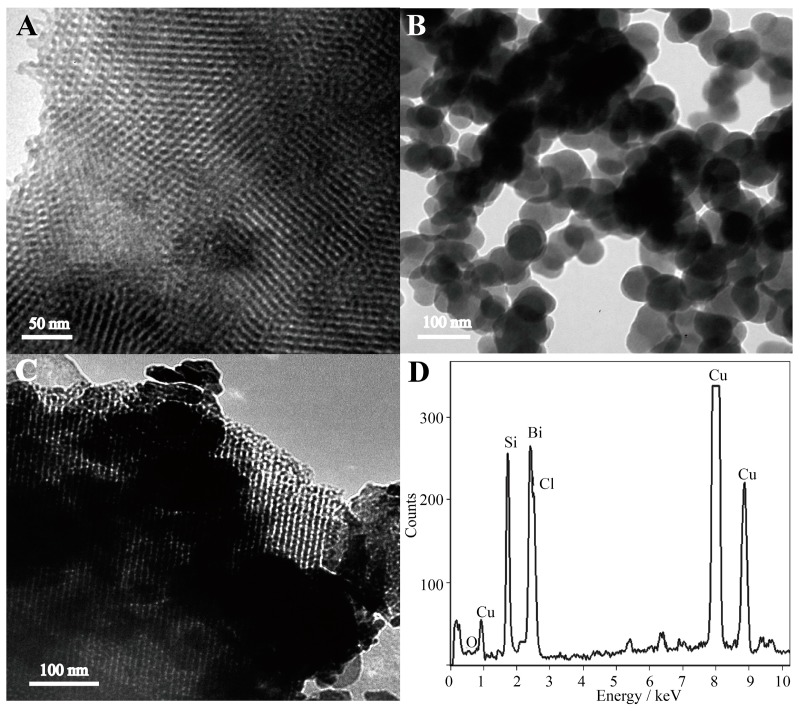
TEM images of the pure (**A**) SiO_2_ KIT-6, (**B**) BiOCl, (**C**) BiOCl-SiO_2_ KIT-6, and (**D**) energy-dispersive X-ray (EDX) spectrum of BiOCl- SiO_2_ KIT-6 composite.

**Figure 3:**
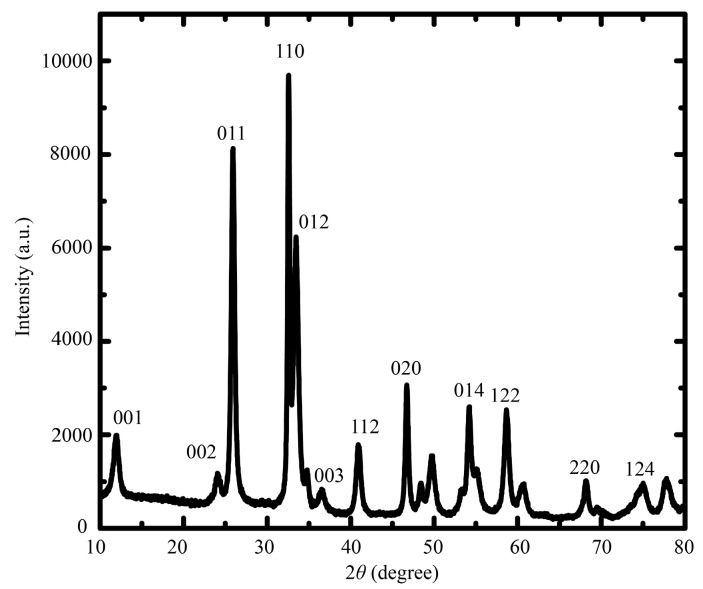
XRD pattern of BiOCl-SiO_2_ KIT-6 composite.

The surface morphologies of prepared samples are shown in Figure 1. SEM images revealed that the obtained sample of BiOCl was composed of microspheres with a diameter of about 3 µm (Figure 1A). The BiOCl-SiO_2_ KIT-6 composites were relatively large, with a distributed size of tens of micrometers (Figure 1B), which displays bunched balls of raspberry-like structures in comparison with the layered assembly of BiOCl. It was found that both BiOCl and BiOCl-SiO_2_ KIT-6 composites were composed of nanoplates of several nanometers in thickness, aligning radically and tightly to form hierarchical microspheres (inset). Notably, these raspberry-like structures of BiOCl with enlarged surface areas may favor the incorporation and transfer of substances. TEM provided additional structural information about the in situ growth of the BiOCl nanoplates in the KIT-6 network. At the edge of the KIT-6 supports, the visible distribution of BiOCl particles was observed in the composites with partitioned light contrast between BiOCl and mesoporous KIT-6 (Figure 2A–C), demonstrating that BiOCl particles may be formed on the KIT-6 mesopores of the supports. Corresponding energy-dispersive X-ray (EDX) spectroscopy was performed on 20 isolated particles using the same sample grid, which indicated the presence of elements of Bi, Si, O, Cl, and Cu (from the grid support) (Figure 2D). X-ray diffraction (XRD) analysis was performed to study the crystallographic structure of the BiOCl-SiO_2_ KIT-6 composites. As shown in Figure 3, the well-crystallized phase of BiOCl-SiO_2_ KIT-6 composite agreed well with that of the tetragonal BiOCl (JCPDS Card No.06-0249). The peaks located at 24.0°, 34.8°, and 36.5° correspond to (002), (012), and (003) crystalline planes of the BiOCl structure, respectively, representing the characteristics of lamellar structures. The crystallite size (average size of the coherent scattering region) for the BiOCl component was found to be 17.8 nm, calculated according to the Scherrer formula, which was comparable with that of 34.4 nm for Mn-doped BiOCl [33], indicating the crystallite formation of BiOCl on the SiO_2_ KIT-6 support.

### 2.2. Electrochemical Characterization

**Figure 4:**
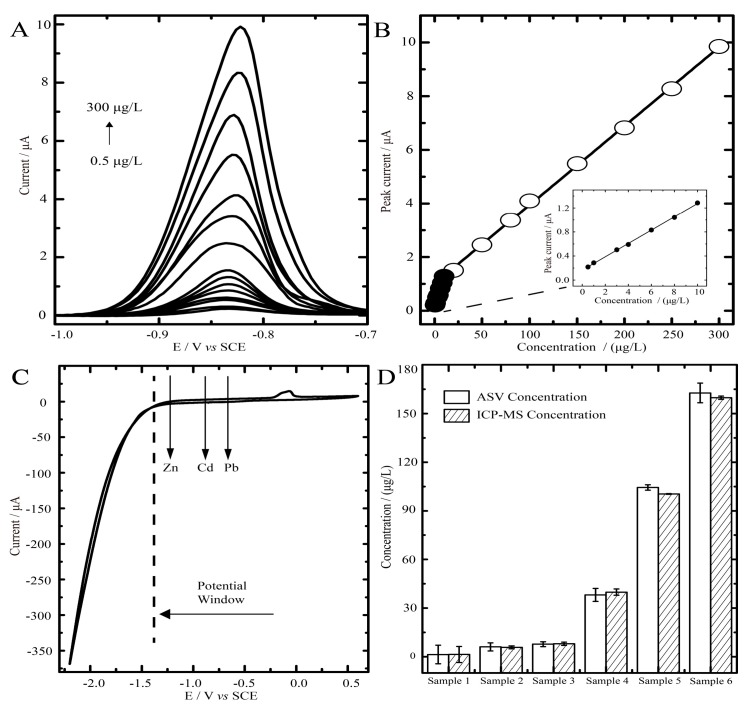
(**A**) Square wave anodic stripping voltammetric (SWASV) responses of peak current on the concentration of Cd^2+^ using BiOCl-SiO_2_ KIT-6/GCE in 0.1 M acetate buffer solution (pH 4.5) at optimal conditions. (**B**) Calibration curves of Cd^2+^ over a concentration range of 0.5 to 300 μg/L. (**C**) Cyclic voltammetric (CV) analysis of BiOCl-SiO_2_ KIT-6/GCE in acetate buffer (0.1 M, pH 4.5). (**D**) The determination result of blood cadmium concentration by anodic stripping voltammetry (ASV) using BiOCl-SiO_2_ KIT-6/GCE in comparison with ICP-MS assay.

Considering the insolubility of the composites in the acetate buffer for anodic stripping voltammetric (ASV) analysis, we performed cyclic voltammetry (CV) in HCl solution to assess the electrochemical behaviors of BiOCl in the composite materials. The recorded voltammograms of the materials exhibit one pair of cathodic and anodic peaks from −0.8 to + 0.6 V *vs.* saturated calomel electrode (SCE) (Figure S1A) with a linear correlation between the peak current (Ip) and the square root of scan rate (*ν*^1/2^), which indicates characteristics of a diffusion-controlled mechanism involved. Using the Randles-Sevcik equation, the diffusion coefficient of bismuth ions (Bi^3+^) of the composites in the HCl solution was calculated to be 3.15 cm^2^/s, close to the previous report [39], which is possibly due to the refined BiOCl assembly favorable for the electron-transfer. For the electrochemical analysis of cadmium, the surface area is an important parameter. To estimate the effective working area of BiOCl-SiO_2_ KIT-6/GCE, we performed cyclic voltammetry (CV) of an electrode in 1 mmol/L K_3_[Fe(CN)_6_] solution containing 0.1 mol/L KCl at 25 °C (diffusion coefficient of K_3_[Fe(CN)_6_] is known to be 7.6 × 10^−6^ cm^2^/s) (Figure S4). Based on the Randles-Sevcik formula, the effective working area of BiOCl-SiO_2_ KIT-6/GCE was calculated to be 0.0294 cm^2^. In addition, we studied the operational potential window of constructed BiOCl-SiO_2_ KIT-6/GCE by recording the CV spectra in 0.1 M acetate buffer. It was observed that BiOCl-SiO_2_ KIT-6/GCE possessed a wide potential window ranging from −1.22 to −0.25 V vs. SCE, which in principle may permit the additional electrochemical discrimination of Zn^2+^ (stripped at −1.15 V) and Pb^2+^ (stripped at −0.58 V) (Figure 4C). 

### 2.3. Anodic Stripping Voltammetric Analysis of Cadmium on BiOCl-SiO_2_ KIT-6/GCEs

Compared with that of bare GCE, the stripping peak current of Cd (II) at the concentration of 100 μg/L by BiOCl-SiO_2_ KIT-6/GCE is significantly higher (Figure S3), demonstrating its ability to perform the electrochemical stripping analysis of cadmium. To best construct the BiOCl-SiO_2_ KIT-6/GCE for cadmium analysis, we firstly optimized the experimental conditions, including the dropping volume of decorated materials, assay pH, deposition potential and time (Figure S2). The optimization results for the determination of Cd^2+^ using BiOCl-SiO_2_ KIT-6/GCE are outlined in Table 1. Under the optimized conditions, the stripping curves of BiOCl-SiO_2_ KIT-6/GCE towards Cd^2+^ determination are shown in Figure 4A. The calibration curves for Cd^2+^ displayed two linear ranges from 0.5 to 10 μg/L and 10 to 300 μg/L, obtained with the regression equations of y = 0.0294x + 1.0085 (R^2^ = 0.9992, [Cd^2+^] > 10 μg/L) and y = 0.1109x + 0.1645 (R^2^ = 0.9990, [Cd^2+^] ≤ 10 μg/L), where y and x are the peak current (μA) and Cd^2+^ concentration (μg/L), respectively (Figure 4B). The wider linear range of cadmium of 0.5–300 μg/L compared with those recorded in earlier reports [25] was achieved by using BiOCl-SiO_2_ KIT-6/GCE. The detection limit was 65 ng/L (S/N = 3), which is 20-fold of one Bi-based electrode [40] and is close to another Bi/multi-walled carbon nanotube-modified electrode [41]. The comparison results regarding Cd (II) determination of our constructed sensor and those previously reported bismuth-based electrodes are briefly summarized in Table 2. Moreover, the BiOCl-SiO_2_ KIT-6/GCE could be repeatedly used for at least 30 continuous times in one day and at least 10 continuous days with a marginal reduction of stripping peak current of 6.8% and 3.6%, respectively.

### 2.4. Blood Samples Determined by ASV on BiOCl—SiO_2_ KIT-6/GCEs and ICP-MS

We further used BiOCl-SiO_2_ KIT-6/GCE for the cadmium determination in cadmium-spiked blood samples. The blood samples were collected from the local hospital following their guidelines. The results from the standard solutions were used as the calibration curve and the cadmium concentration could be calculated from the measured current signals. We defined these concentrations as ASV concentrations, which were compared with ICP-MS results. As shown in Figure 4D, good agreement between two methods was achieved in six samples (relative error from −4.26% to +4.64%), indicating that the constructed BiOCl-SiO_2_ KIT-6/GCE for the ASV determination of blood cadmium is effective and reliable, yet more convenient than the alternative method.

## 3. Materials and Methods

### 3.1. Chemicals and Solutions

Bismuth nitrate pentahydrate, tetraethyl orthosilicate (TEOS), potassium chloride, potassium ferricyanide, sodium acetate, acetic acid, hydrochloric acid, perfluorinated sulfonic acid ester (Nafion), sodium chloride and ethylene glycol (EG) were purchased from the Sinopharm Chemical Reagent Co., Ltd. (Shanghai, China). A triblock copolymer, poly(ethylene glycol)-block-poly(propylene glycol)-block-poly(-ethylene glycol) (Pluronic P123 with a molecular weight of 5800, EO_20_PO_70_EO_20_) was obtained from Sigma-Aldrich (ST Louis, MO, USA) and used as the structure-directing template. N-butyl alcohol was purchased from Aladdin (Shanghai, China). The stock solutions of Cd^2+^, Pb^2+^, and Zn^2+^ (1000 mg/L in 5 wt. % HNO_3_) were purchased from NACIS (Qingdao, China). All chemical reagents were of analytical grade. 

### 3.2. Instrumentation

Electrochemical data were obtained by an electrochemical analyzer (CHI760D, Shanghai, China) with a three-electrode system, containing a glassy carbon working electrode (Φ = 3 mm), a platinum electrode and a saturated calomel electrode (SCE) as a reference. The microscopic features of the samples were recorded on a scanning electron microscope (SEM, Zeiss Supra55, Heidenheim Germany) operated at an accelerating voltage of 5.00 kV. Transmission electron microscopy (TEM) images were recorded with an electron microscope (JEOL-1010, Tokyo, Japan) operated at 200 kV. Wide-angle X-ray diffraction (XRD) pattern was collected on a Rikagu Smartlab TM 9 KW X-ray diffractometer (Rigaku Corporation, Tokyo, Japan) in the 2*θ* from 10–80° using Cu K*α* radiation. Inductively coupled plasma mass spectrometry (ICP-MS) analysis was obtained by a Thermo Scientific iCAP Q instrument (Waltham, MA, USA).

### 3.3. Synthesis of Mesoporous Silica KIT-6

KIT-6 with a 3D-cubic structure was synthesized as reported [42]. Typically, 6.0 g of P123 was dissolved in 217.0 g ultrapure water and 11.8 g of HCl (35%) solution. After the solution became homogeneous, 6.0 g of n-butanol was added to the solution and mixed for 1 h. 12.9 g of TEOS was added to the solution and mixed for another 24 h. For all the procedures, the reaction temperature was maintained at 36 °C. The product was transferred in a Teflon autoclave and hydrothermally treated at 100 °C for 24 h under a static condition. The final solution was filtered and dried at 100 °C for 24 h in an air oven and the organic surfactant was removed by calcination at 550 °C for 6 h.

### 3.4. Synthesis of BiOCl-SiO_2_ KIT-6 Composites

A facile, *in situ* hydrolysis method under hydrothermal conditions was used to synthesize the BiOCl-SiO_2_ KIT-6 composites. Typically, 1.5 mmol of Bi(NO_3_)_3_·5H_2_O was fully dissolved in 30 mL of EG under stirring at room temperature. Subsequently, 0.2 mmol SiO_2_ KIT-6 was added to the above solution. The mixture was further stirred for 30 min at room temperature before the addition of 10 mmol of NaCl. Then, the mixture was transferred into a Teflon-lined stainless steel autoclave (Shandong, China) and maintained at 170 °C for 6 h. After the reaction was completed, the autoclave was allowed to cool down and a product of white powder was obtained. Finally, the product was centrifuged and washed with distilled water several times and then dried at 60 °C for 5 h. 

### 3.5. Preparation of BiOCl-SiO_2_ KIT-6 Modified GCE

GCE was first polished with 0.3 μm alumina slurries on a polishing pad, then rinsed with ultrapure water and sonicated thoroughly. The treated GCE was evaluated in the buffer solution containing 1 mmol/L K_3_[Fe(CN)_6_] and 0.1 mol/L KCl by cyclic voltammetry. Then the BiOCl-SiO_2_ KIT-6/GCEs were prepared by the drop-coating of an appropriate amount of materials on the polished GCE surface. Briefly, 1 mg of either synthesized BiOCl or BiOCl-SiO_2_ KIT-6 was dispersed in 1 mL of 0.1% (*v/v*) Nafion using a sonicator. Then, 10 μL aliquots of the suspensions were dropped on the GCE surface and dried at room temperature. The permselective membrane of Nafion was used due to its ability to reduce the interference of surface-active compounds but not to impede the mass transport of analytes for the stripping analysis [43]. 

### 3.6. Electrochemical Measurements

Square wave anodic stripping measurements were performed with a deposition of cadmium using BiOCl-SiO_2_ KIT-6/GCE as the working electrode. The deposition potential was applied to working electrodes while the solution was stirred. After the deposition period, the assay buffer stood by for 20 s, and then the stripping process was recorded by using square-wave voltammetry in the potential range from −1.0 to −0.3 V vs. SCE. Before the next run, a cleaning step was applied by holding the work electrode at the potential of +0.3 V for 30 s.

Cyclic voltammetry (CV) measurements were performed using BiOCl-SiO_2_ KIT-6/GCE in 1 mol/L HCl at different scan rates of 25, 50, 100, 150 and 200 mV/s. For cyclic voltammetry, the potential was swept from −0.8 to +0.6 V vs*.* SCE. All experiments were carried out at room temperature (25 ± 2 °C).

### 3.7. Pretreatment of Blood Samples and ICP-MS Measurement

Human blood samples were obtained from affiliated hospitals of Nanjing Medical University. The study is approved by the Institutional Review Board for Human Studies of Nanjing Medical University and informed consent was obtained. All experimental protocols were in compliance with all government policies and defined protocols. The human whole blood samples were treated based on the U.S. EPA (Environmental Protection Agency) Method 3050B with some modifications. Typically, 0.5 mL of whole blood spiked with a certain amount of standard cadmium solution was firstly applied to a 5 h digestion with 10 mL concentrated HNO_3_ at 105 °C until close to dryness, followed by a dilution to 2 mL using 1% (*v/v*) HNO_3_. The concentration of cadmium in the treated sample was measured by ICP-MS (Thermo Scientific iCAP Q, Waltham, MA, USA) using indium isotope (^114^In) as an internal calibration standard. For the electrochemical stripping analysis, the samples were diluted to 2 mL with 0.1 M acetate buffer (pH 4.5).

## 4. Conclusions

In conclusion, the BiOCl-SiO_2_ KIT-6 composites with a raspberry-like surface structure of BiOCl were facilely prepared through an in situ synthesis method under hydrothermal conditions. A novel BiOCl-based mesoporous silicon composite-modified sensor for the electrochemical stripping analysis of cadmium, applicable for the analysis of cadmium-spiked real blood samples, was thus successfully constructed. The improved electrochemical performance of the constructed BiOCl-SiO_2_ KIT-6/GCE may be attributed to the refined and modulated microstructure of BiOCl on the siliceous support. The developed sensor possessed a wide operational potential window, permitting the detection of up to three typical heavy metals and a remarkable sensitivity to cadmium with a wide linear concentration range. As the mesoporous structural support of KIT-6 attributed to the surface morphology of loaded BiOCl is essential for the electrochemical performance of the composites, further work on the ordered mesostructure of constructed bismuth oxychloride materials linked with photo-catalytic/-electrochemical properties will be conducted for extreme conditions applications, i.e., complicate toxic waste waters containing heavy metal ions and other organic pollutants, as well as for the simultaneous detection of several heavy metals.

## Figures and Tables

**Table 1:** Analytical parameters for ASV in the determination of Cd^2+^ obtained by BiOCl-SiO_2_ KIT-6/GCE.

Electrode	pH	Deposition Potential (V)	Deposition Time (s)	Dropping Volume of Composite (μL)
BiOCl-SiO_2_ KIT-6/GCE	4.5	−1.3	120	10

**Table 2:** Comparison of the analytical performance of Bi film-modified electrodes for the measurement of Cd (II) by ASV.

Electrode	Linear Range (μg/L)	Detection Limit (μg/L)	Reference
Bi/SPME	NR	1.3	[40]
Bi/MWCNT-EBP-NA/GCE	1.0–50.0	0.06	[41]
BiOCl/MWCNT/GCE	5–50	1.2	[26]
BiFE	NR	0.2	[15]
BiOPE	20–100	1.5	[30]
BiOCl-SiO_2_ KIT-6/GCE	0.5–300	0.065	This work

Bi/SPME: bismuth-coated screen-printed microband electrode, Bi/MWCNT-EBP-NA/GCE: Bi/multi-walled carbon nanotube-emeraldine base polyaniline-Nafion composite-modified glassy carbon electrode, BiOCl/MWCNT/GCE: BiOCl particle-multiwalled carbon nanotube composite-modified glassy carbon electrode, BiFE: bismuth film electrode, BiOPE: bismuth oxide printed on electrode, NR: not reported.

## Supplementary Materials

The following are available online at www.mdpi.com/1420-3049/22/5/797/s1.
