# Supplementary material for: Raspberry-Like Bismuth Oxychloride on Mesoporous Siliceous Support for Sensitive Electrochemical Stripping Analysis of Cadmium
Source: Molecules. 2017 May 13;22(5):797. doi: 10.3390/molecules22050797 (PMC6154545; doi:10.3390/molecules22050797)
Supplement: Supplementary file 1 [file molecules-22-00797-s001.pdf]

Supplementary information

Raspberry-Like Bismuth Oxychloride on Mesoporous Siliceous Support for Sensitive Electrochemical Stripping Analysis of Cadmium

Yiyan Song[1], Zhihui Xu[1], Xinyu Yu[1], Xueyan Shi[2], Huijun Jiang[2], Xiaoming Li[3], Yan Kong[3], Qin Xu[4] and Jin Chen [1,5,*]

[1] School of Public Health, Nanjing Medical University, 211166 Nanjing, China.

[2] School of Pharmacy, Nanjing Medical University, 211166 Nanjing, China.

[3] State Key Laboratory of Materials-Oriented Chemical Engineering, College of Chemistry and Chemical Engineering, Nanjing University of Technology, 211166 Nanjing, China.

[4] College of Chemistry and Chemical Engineering, Yangzhou University, Yangzhou 225002, China

[5] The Key Laboratory of Modern Toxicology, Ministry of Education, School of Public Health, Nanjing Medical University, 210093 Nanjing, China.

Correspondence and request for materials should be addressed to J.C. (email: okachen30@gmail.com; jchen@njmu.edu.cn)

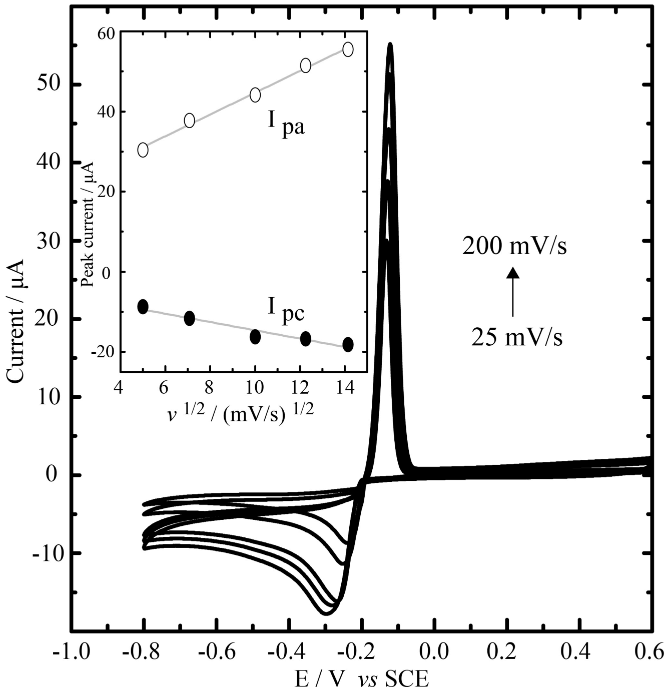

Figure S1. Cyclic voltammograms of a glassy carbon electrode in 1 mol/L HCl containing BiOCl-SiO_2 KIT-6 composite (50 mg/L) at the different scan rates of 25, 50, 100, 150 and 200 mV/s. The inset shows the linear relationship between the peak current (I_p) and the square root of the scan rate ($v^{1/2}$).

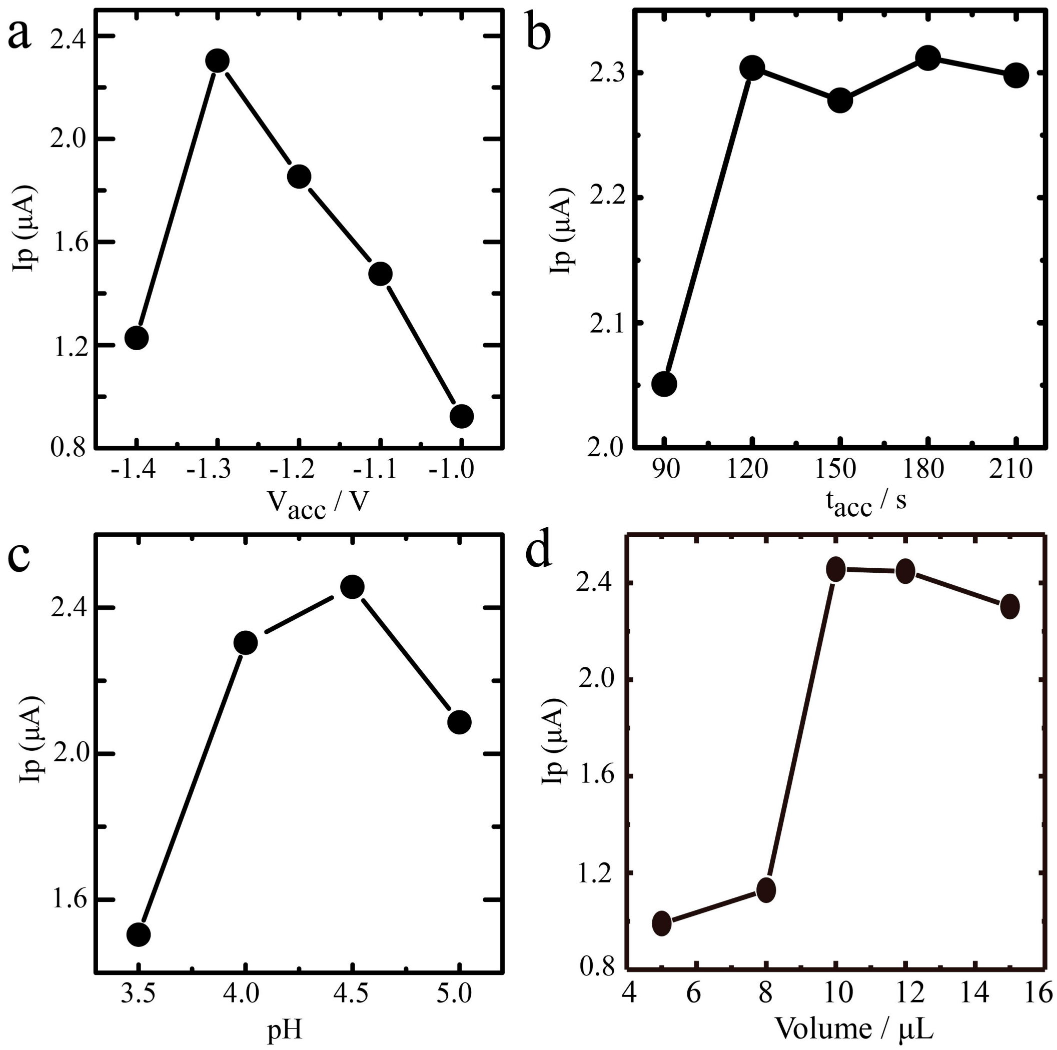

Figure S2. Square wave anodic stripping voltammetry (SWASV) responses of 50 μg/L of Cd^{2+} on BiOCl-SiO_2 KIT-6/GCE with (a) different deposition potentials from -1.0 - -1.4 V (0.1 M acetate buffer pH 4.0; deposition time 120 s; 10 μL of BiOCl-SiO_2 KIT-6 composite suspension-modified GCE); (b) different deposition times from 90 - 210 s (0.1 M acetate buffer pH 4.0; deposition potential -1.3 V; 10 μL of BiOCl-SiO_2 KIT-6 composite suspension-modified GCE); (c) different pH levels from 3.5 - 5.0 in 0.1 M acetate buffer solution (deposition potential -1.3 V; deposition time 120 s; 10 μL of BiOCl-SiO_2 KIT-6 composite suspension-modified GCE); and (d) different volumes of BiOCl-SiO_2 KIT-6 composite suspension (1 mg/mL) from 5-15 μL used to modify the GCE (deposition potential -1.3 V; deposition time 120 s; 0.1 M acetate buffer pH 4.5).

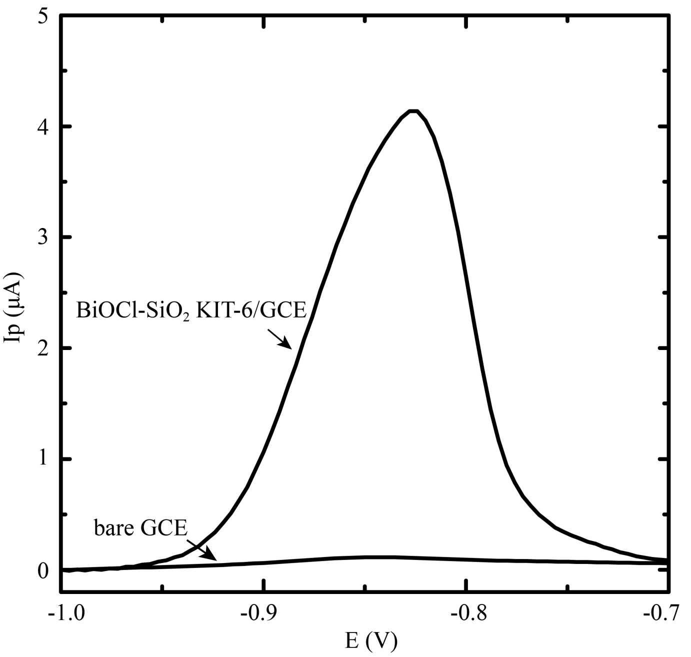

Figure S3. SWASV responses of Cd^{2+} of the concentration of 100 μg/L on BiOCl-SiO_2 KIT-6/GCE and bare GCE.

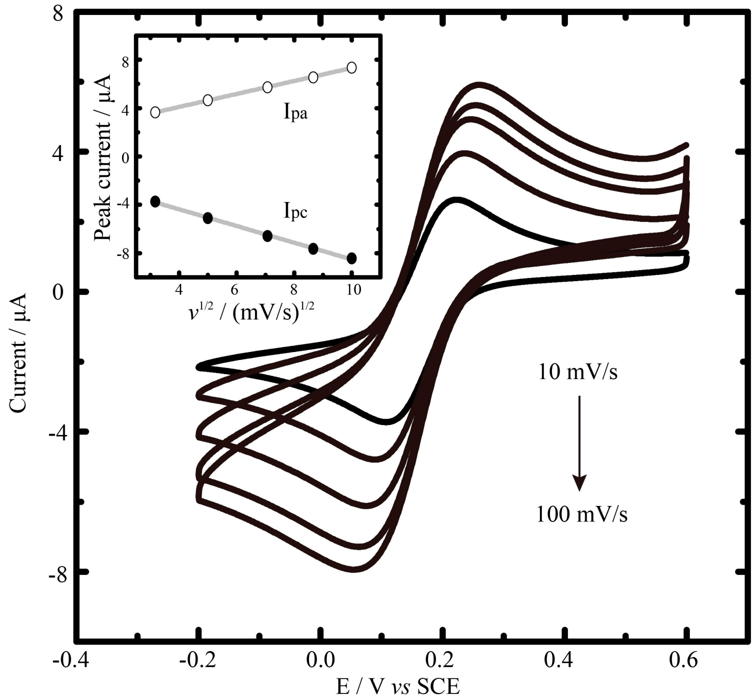

Figure S4. Cyclic voltammograms of BiOCl-SiO_2 KIT-6/GCE in 1 mmol/L $K_3[Fe(CN)_6]$ solution containing 0.1 mol/L KCl at the different scan rates of 10, 25, 50, 75 and 100 mV/s. The inset shows the linear relationship between the peak current (I_p) and the square root of the scan rate ($v^{1/2}$).
